# New Clinical Strain of *Neisseria gonorrhoeae* with Decreased Susceptibility to Ceftriaxone, Japan

**DOI:** 10.3201/eid2201.150868

**Published:** 2016-01

**Authors:** Takashi Deguchi, Mitsuru Yasuda, Kyoko Hatazaki, Koji Kameyama, Kengo Horie, Taku Kato, Kohsuke Mizutani, Kensaku Seike, Tomohiro Tsuchiya, Shigeaki Yokoi, Masahiro Nakano, Mutsumasa Yoh

**Affiliations:** Gifu University, Gifu, Japan (T. Deguchi, M. Yasuda, K. Hatazaki, K. Kameyama, K. Horie, T. Kato, K. Mizutani, K. Seike, T. Tsuchiya, S. Yokoi, M. Nakano);; Yoh Clinic, Inazawa, Japan (M. Yoh)

**Keywords:** Neisseria gonorrhoeae, ceftriaxone, gonorrhea, Japan, bacteria, antimicrobial resistance, sexually transmitted infections, pharyngeal gonorrhea, acute urethritis

**To the Editor:** In 2009, 2010, and 2013, *Neisseria gonorrhoeae* strains H041 (ceftriaxone MIC of 2 mg/L), F89 (ceftriaxone MIC of 1 mg/L), and A8806 (ceftriaxone MIC of 0.5 mg/L) were isolated from samples from patients in Japan (*1*), France (*2*) and Australia (*3*), respectively. In Japan, no other clinical *N.* *gonorrhoeae* strains with decreased susceptibility to ceftriaxone were reported until 2014, when clinical strain GU140106 (ceftriaxone MIC of 0.5 mg/L) was isolated from a man in in Nagoya, Japan. We report details of this case and sequencing results of the *penA* gene for the strain. The study was approved by the Institutional Review Board of the Graduate School of Medicine, Gifu University, Japan.

*N. gonorrhoeae* strain GU140106 was isolated from a urethral swab sample from a man with acute urethritis. The man had received fellatio, without condom use, from a female sex worker in Nagoya in December 2013. He visited our clinic in January 2014 for urethral discharge. Culture of a urethral swab sample was positive for *N. gonorrhoeae*. We used the Cobas 4800 CT/NG Test (Roche Molecular Systems Inc., Pleasanton, CA, USA) to test a first-voided urine sample; results were positive for *N. gonorrhoeae* but negative for *Chlamydia trachomatis*. The infection was treated with a single-dose regimen of ceftriaxone (1 g) administered by intravenous drip infusion. Two weeks later, the man reported no symptoms, and his first-voided urine sample was negative for leukocytes. The test-of-cure for *N. gonorrhoeae* was not performed. The female sex worker could not be examined for the presence of *N. gonorrhoeae* strain GU140106 in her pharynx.

The strain was confirmed to be a gonococcal species by testing with Gonochek-II (TCS Biosciences Ltd, Buckingham, UK), the HN-20 Rapid system identification test (Nissui, Tokyo, Japan), and the Aptima Combo 2 assay for CT/NG (Hologic, Inc., Bedford, MA, USA) and by 16S rRNA gene sequencing and *porA* pseudogene PCR (*4*). MICs of antimicrobial drugs for GU140106 were as follows, as determined by using the agar dilution method: 2.0 mg/L for penicillin G, 1.0 mg/L for tetracycline, 2.0 mg/L for cefixime, 0.5 mg/L for ceftriaxone, 8.0 mg/L for levofloxacin, 0.5 mg/L for azithromycin, and 32.0 mg/L spectinomycin. The strain was determined to be resistant to penicillin G, tetracycline, cefixime, ceftriaxone, and levofloxacin, according to criteria of the European Committee on Antimicrobial Susceptibility Testing (*5*)

The *penA* gene of strain GU140106 was sequenced as previously described (*6*); results showed the presence of a novel mosaic penicillin-binding protein 2 (PBP2; GenBank accession no. LC056026) (Figure). Multilocus sequence typing (MLST) and *N. gonorrhoeae* multiantigen sequence typing (NG-MAST) of GU140106 were performed as previously reported (*8*,*9*). MLST assigned strain GU140106 to sequence type 7363, the same as strains H041 and A8806 (*1*,*2*). NG-MAST assigned strain GU140106 to sequence type 6543. MLST and NG-MAST results for GU140106 differed from those for F89 (*3*).

**Figure:**
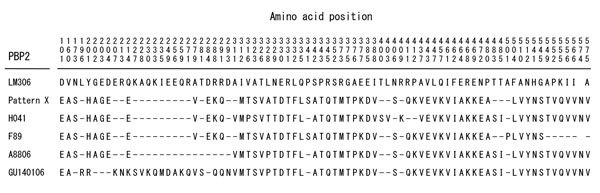
Sequences of altered amino acids in penicillin-binding protein 2 (PBP2) of *Neisseria gonorrhoeae* strains with decreased susceptibility to oral cephalosporins and strains with resistance to ceftriaxone. Strain GU140106 was isolated from a urethral swab sample from a man in in Nagoya, Japan, who had received fellatio, without condom use, from a female sex worker. Sequences are aligned with wild-type PBP2 derived from nucleic acid sequence of the *penA* gene of penicillin-susceptible *N. gonorrhoeae* strain LM306 (GenBank accession no. M32091). The PBP2 pattern X of strains with decreased susceptibility to oral cephalosporins is quoted from our previous study (*6*). The PBP2s of ceftriaxone-resistant strains H041 and F89 are derived from their *penA* genes (GenBank accession nos. AB546858 and JQ073701, respectively). The PBP2 of strain A8806, which has decreased susceptibility to ceftriaxone, is derived from the nucleic acid sequence of the *penA* gene (David M. Whiley, pers. comm., 2015). In strain H041, the concurrent presence of substitutions A311V, V316P, and T483S was reported to be responsible for conferring resistance to ceftriaxone (*7*). The PBP2 of strain GU140106 is derived from the nucleic acid sequence of the *penA* gene (GenBank accession no. LC056026); the strain has the same A311V and T483S substitutions as strain H041, but it has substitution V316T instead of V316P. The PBP2s of strains GU140106 and A8806 have the same amino acid substitutions at A311, V316, and T483. In addition, PBP2 of strain GU140106 has several amino acid changes in positions 227–281 that were not observed in other strains.

Since the naming of the mosaic PBP2 associated with decreased susceptibilities to oral cephalosporins as pattern X (*6*), various PBP2 mosaic structures have been discovered. Mosaic PBP2 structures are basically composed of fragments analogous to PBP2s in *Neisseria* species. Before strain H041 emerged, strains harboring mosaic PBP2s had been resistant to oral cephalosporins but susceptible to ceftriaxone. H041 (ceftriaxone MIC of 2 mg/L) had additional novel amino acid changes, including A311V, V316P, and T483S, in its mosaic PBP2. The presence of substitutions A311V, V316P, and T483S was reported to be responsible for resistance to ceftriaxone (*7*). Like strain H041, strains GU140106 and A8806 (ceftriaxone MICs of 0.5 mg/L) had substitutions A311V and T483S, but instead of substitution V316P, they had substitution V316T. In addition, GU140106 had several changes in positions 227–281 that were not present in other strains. These alterations might also contribute to the decreased susceptibility to ceftriaxone.

On the basis of pharmacodynamic analyses (*10*), a 1-g dose of ceftriaxone (the recommended first-line treatment for gonorrhea in Japan) would be effective against genital gonorrhea caused by strains exhibiting decreased susceptibility to ceftriaxone (e.g., strains GU140106 and A8806). However, such strains could be resistant to lower-dose regimens, including 250-mg and 500-mg doses of ceftriaxone.

This *N. gonorrhoeae* strain, GU140106, was isolated from the urethra of a man who received fellatio from a female sex worker; thus, the bacteria could have derived from her pharynx. *N. gonorrhoeae* strain H041 was previously isolated from the pharynx of a female sex worker (*1*). To prevent the emergence and spread of ceftriaxone-resistant *N. gonorrhoeae*, pharyngeal gonorrhea must be treated. It is uncertain whether a 1-g dose of ceftriaxone would be effective against pharyngeal gonorrhea caused by strains with decreased susceptibility to ceftriaxone, and this regimen might facilitate the selection of such strains from oral cephalosporin-resistant strains in the pharynx. The emergence of *N. gonorrhoeae* GU140106 in Japan suggests that new strategies (not just increased ceftriaxone doses), including combination treatment with ceftriaxone and another class of antimicrobial drugs and multiple dose regimens of ceftriaxone, might be required to treat pharyngeal gonorrhea.
